# Molecular evolution of the insect-specific flaviviruses

**DOI:** 10.1099/vir.0.036525-0

**Published:** 2012-02

**Authors:** Shelley Cook, Gregory Moureau, Andrew Kitchen, Ernest A. Gould, Xavier de Lamballerie, Edward C. Holmes, Ralph E. Harbach

**Affiliations:** 1 Natural History Museum, Cromwell Road, London SW7 5BD, UK; 2 Unité des Virus Emergents UMR190, Université de la Méditerranée, Institut de Recherche pour le Développement, EHESP French School of Public Health, Marseille, France; 3 Center for Infectious Disease Dynamics, Department of Biology, The Pennsylvania State University, University Park, PA 16802, USA; 4 Centre for Ecology and Hydrology, Wallingford, Oxfordshire OX10 8BB, UK; 5 Fogarty International Center, National Institutes of Health, Bethesda, MD 20892, USA

## Abstract

There has been an explosion in the discovery of ‘insect-specific’ flaviviruses and/or their related sequences in natural mosquito populations. Herein we review all ‘insect-specific’ flavivirus sequences currently available and conduct phylogenetic analyses of both the ‘insect-specific’ flaviviruses and available sequences of the entire genus *Flavivirus*. We show that there is no statistical support for virus–mosquito co-divergence, suggesting that the ‘insect-specific’ flaviviruses may have undergone multiple introductions with frequent host switching. We discuss potential implications for the evolution of vectoring within the family *Flaviviridae*. We also provide preliminary evidence for potential recombination events in the history of cell fusing agent virus. Finally, we consider priorities and guidelines for future research on ‘insect-specific’ flaviviruses, including the vast potential that exists for the study of biodiversity within a range of potential hosts and vectors, and its effect on the emergence and maintenance of the flaviviruses.

## Introduction

The genus *Flavivirus* contains many important human pathogens causing haemorrhagic fever and encephalitis, such as dengue virus (DENV), yellow fever virus (YFV) and Japanese encephalitis virus (JEV). Flaviviruses may be (i) arthropod-borne, infecting a range of vertebrate hosts through mosquito or tick bites, (ii) presumed to be limited to vertebrates alone or (iii) apparently limited to insects alone. The last group, referred to as the ‘insect-specific’ flaviviruses, contains tentative members of the genus *Flavivirus* that appear to replicate only in mosquito cells and not in mammalian cells (Kuno, 2007).

Cell fusing agent virus (CFAV) is an ‘insect-specific’ flavivirus that was first discovered over 30 years ago during laboratory studies, when cytopathic effect was observed in a *Stegomyia albopicta* ( = *Aedes albopictus*) cell culture following addition of supernatant medium from a *Stegomyia aegypti* ( = *Aedes aegypti*) cell line (Stollar & Thomas, 1975). However, the genomic sequence of the virus was not characterized for a further 17 years (Cammisa-Parks *et al.*, 1992). Subsequently, CFAV was listed by the International Committee on Taxonomy of Viruses (ICTV) as a tentative member of the genus *Flavivirus* (Fauquet & Fargette, 2005). The first isolations of ‘insect-specific’ flaviviruses from natural mosquito populations were reported recently and included the isolation of CFAV from Puerto Rico and Kamiti River virus (KRV) from Kenya (Cook *et al.*, 2006; Crabtree *et al.*, 2003; Sang *et al.*, 2003). Even more recently, *Culex* flavivirus (CxFV) has been isolated and characterized from *Culex* mosquitoes in Japan, Guatemala, Mexico, Uganda, the USA and Trinidad and Tobago (Cook *et al.*, 2009; Farfan-Ale *et al.*, 2009, 2010; Hoshino *et al.*, 2007; Kim *et al.*, 2009; Morales-Betoulle *et al.*, 2008), and *Aedes* flavivirus (AeFV) from *Stegomyia flavopicta* ( = *Aedes flavopictus*) and *St. albopicta* mosquitoes from Japan (Hoshino *et al.*, 2009). In addition, Quang Binh virus (QBV) from *Cx. tritaeniorhynchus* in Vietnam (Crabtree *et al.*, 2009) and Nakiwogo virus (NAKV) isolated from *Mansonia africana nigerrima* mosquitoes from Uganda (Cook *et al.*, 2009) are tentative members of the insect-specific group. The most recent phylogenetic trees of the ‘insect-specific’ flaviviruses imply the divergence of two groups, reflecting sequences isolated from *Stegomyia* ( = *Aedes*) versus *Culex* mosquitoes (Hoshino *et al.*, 2009), and there is evidence for vertical transmission, with CFAV isolated from both male and female mosquitoes from a range of species (Cook *et al.*, 2006).

Theoretical studies have estimated the existence of over 2000 undiscovered mosquito-borne flaviviruses (Pybus *et al.*, 2002), and there has been a recent explosion in the number and diversity of sequences that appear to be related to ‘insect-specific’ flaviviruses amplified from mosquitoes (Aranda *et al.*, 2009; Bolling *et al.*, 2011; Calzolari *et al.*, 2010; Farfan-Ale *et al.*, 2009, 2010; Kihara *et al.*, 2007; Morales-Betoulle *et al.*, 2008; Pabbaraju *et al.*, 2009; Roiz *et al.*, 2009; Sánchez-Seco *et al.*, 2010). Flavivirus RNA has also recently been discovered in phlebotomine sandflies from Algeria (Moureau *et al.*, 2010). However, in many cases, none of (i) molecular identification of the mosquito species, (ii) de-pooling to test individual specimens or (iii) isolation in cell culture has been carried out. This is significant because results may be confounded by DNA sequences related to flaviviruses that have been discovered in the genomes of *St. aegypti* and *St. albopicta*. These sequences probably resulted from integration events following infection of each mosquito species by a virus (or viruses) related to the CFAV group (Crochu *et al.*, 2004). In addition, there is differing taxonomic coverage in available flaviviral sequences with respect to virus strain and/or gene regions. From one tentative member of this group listed in 2002, namely CFAV, there are now well over 60 different E gene region sequences deposited in GenBank (www.ncbi.nlm.nih.gov) that may be related to the ‘insect-specific’ flaviviruses.

Whilst there is clearly a high prevalence and biodiversity of ‘insect-specific’ flaviviruses in nature, our understanding of the significance of the group and implications for the evolution and transmission of viruses belonging to the genus *Flavivirus* is currently limited. With this in mind, we herein review all relevant ‘insect-specific’ sequences currently available, and perform phylogenetic analyses of both the ‘insect-specific’ flaviviruses and all viruses within the genus. Additionally, tests for (i) evidence of recombination events in the history of the group and (ii) potential virus–mosquito co-divergence analyses were also conducted.

## Analyses

### NS5, NS3 and E gene region nucleotide datasets

In general, two datasets were prepared for each gene region of the flaviviruses. First, an ‘insect-specific focus’ dataset was prepared, which was limited to ‘insect-specific’ flavivirus taxa plus three outgroup taxa, namely tick-borne encephalitis virus, Rio Bravo virus and DENV. Second, a ‘global genus’ dataset was prepared containing all available taxa from across the genus *Flavivirus* for that gene. Hence, for regions encoding the NS5 and NS3 proteins, both an ‘insect-specific focus’ and a ‘global genus’ dataset were prepared. In general, the ‘insect-specific focus’ dataset contained a larger number of ‘insect-specific’ sequences, but of a relatively shorter length than the ‘global genus’ dataset, reflecting sequence availability in public databases. For all nucleotide datasets, alignment was conducted by using muscle on deduced amino acids (Cook & Holmes, 2006; Edgar, 2004), and nucleotide sequences were aligned using this amino acid guide alignment. GenBank accession numbers for all sequences analysed are included in phylogenetic trees.

For the NS5 region, there is significant variation in the taxonomic coverage of ‘insect-specific’ sequences available in public databases due to differences in position along the viral genome of primer pairs used for various studies. Hence, to take account of all currently available sequences, a number of datasets were prepared that effectively comprised a ‘sliding window’ along the NS5 gene (data not all shown). This resulted in six nucleotide alignments of varying length, strain composition and number of taxa. Wherever possible, a section of the CSA2 sequence from the *St. aegypti* A20 cell line, which is shared with RNA flaviviral-like sequences found in phlebotomine sandflies, was also included (Crochu *et al.*, 2004; Moureau *et al.*, 2010). For the NS5 ‘global genus’ analysis, the nucleotide dataset contained 76 taxa. In contrast, the ‘insect-specific focus’ dataset for the region encoding the NS3 gene comprised 13 flaviviral strains plus the three outgroup sequences, whereas for the NS3 ‘global genus’ analysis, sequences for 78 flaviviral taxa were available. To estimate phylogenetic trees for the NS3 region that were directly comparable with those from the NS5 region for tests of tree topology as described below, equivalent analyses were conducted for a 76-sequence NS3 dataset that contained exactly the same taxa as the NS5 dataset.

Sixty sequences were available for the E gene ‘insect-specific focus’ analyses. No mosquito-borne, tick-borne or NKV sequences were included as outgroups due to high levels of divergence and ambiguous alignment; hence, trees were midpoint-rooted and a ‘global analysis’ was not conducted. To check the effect of alignment algorithm on this divergent dataset, both clustal (Thompson *et al.*, 1994) and muscle (Edgar, 2004) were used to align sequences, and analyses were conducted on both alignments.

For each single gene nucleotide dataset (both ‘insect-specific focus’ and ‘global genus’ data), all analyses were repeated on datasets constructed with third codon positions excluded and on amino acid translations, with the exception of the highly conserved NS5 ‘insect-specific focus’ alignment. In addition, for the most divergent datasets (namely the NS3 ‘global genus’ and the E gene ‘insect-specific focus’ datasets), alignments were submitted to the GBlocks program, which objectively eliminates poorly aligned positions and divergent regions (Castresana, 2000). Each dataset was treated with (i) default (stringent) and (ii) least stringent settings in GBlocks, producing additional datasets and resultant phylogenies to explore the effect of potential short regions of misalignment.

### ORF dataset

For the analysis of all 76 available flaviviral ORF amino acid sequences, alignment was conducted via muscle. Alignments were subjected to GBlocks stripping (i.e. removal of regions of ambiguous alignment) with both (i) least stringent and (ii) most stringent settings to produce additional datasets for comparison of the effect of short regions of high divergence. An equivalent analysis was conducted with the addition of the following members of the family *Flaviviridae* as outgroup taxa: border disease virus, classical swine fever virus (previously called hog cholera virus), bovine viral diarrhea virus types 1 and 2 and GB virus C. Tamana bat virus (TABV), which is a tentative member of the genus *Flavivirus*, was also included in the *Flaviviridae* analysis. TABV was not included in any other analyses due to its highly divergent nature and ambiguous alignment.

### Phylogenetic analyses

For nucleotide-based analyses, modeltest (Posada & Crandall, 1998) was used to select the best-fit model of nucleotide substitution (the GTR+Γ_4_+I model), and subsequent phylogenetic analyses were conducted using this nucleotide-substitution model under the Bayesian Markov chain Monte Carlo method implemented in MrBayes v3.1.2 (Huelsenbeck & Ronquist, 2001). Equivalent amino acid phylogenetic analyses in MrBayes were conducted using the WAG model of amino acid replacement. All parameters were estimated from the data under default priors. Markov chains were run for a minimum of 20 million generations and the first 10 % of samples were discarded as burn-in, with the exception of the ORF dataset, which was run for 50 million generations. Support for nodes was assessed using posterior probability values calculated in MrBayes. All phylogenetic analyses were carried out on the freely available Bioportal server (http://www.bioportal.uio.no). Stationarity was assessed as effective sample sizes >400 using Tracer v1.4.1 (Drummond & Rambaut, 2007).

### Topology tests

For the ‘global genus’ phylogenies resulting from analyses of the NS3 and NS5 regions and the ORF, an analysis of phylogenetic congruence among the 76-taxa tree topologies was conducted. First, for each single gene region or the ORF, the likelihoods of the maximum posterior probability (MAP) tree and the maximum clade credibility (MCC) tree from the datasets described above were compared by using the SH test in paup* (Swofford, 2003). Specifically, for the NS5 and NS3 nucleotide datasets, the input treefile comprised the MAP and MCC trees produced using (i) the full nucleotide dataset, (ii) with third codon positions removed, and (iii) an amino acid alignment. For the ORF dataset, the input treefile comprised trees produced from the muscle amino acid alignment plus those produced using GBlocks stripping using both the default and least stringent settings. All substitution parameters were estimated from the data. The tree topologies with the highest likelihood for either the NS5 and NS3 regions or for the ORF, respectively, were then compared with all three original datasets, namely the NS5 and NS3 nucleotide alignments and the ORF amino acid alignment (in which nucleotides had been aligned using an amino acid guide alignment). All results are included in Supplementary Table S1.

### Recombination tests

Due to variation in the position of CFAV in the E gene phylogeny compared with the NS3, NS5 and ORF phylogenies (see Results), we assessed whether recombination had occurred within the ‘insect-specific’ flaviviruses. For this analysis, ORF sequences were aligned as amino acids, and nucleotides were aligned using the amino acid alignment guide (length, 10 461 nt). We also (i) trimmed the alignment at both ends (final length 9894 nt) and (ii) deleted regions of ambiguous alignment (8835 nt). All manual adjustments to nucleotide data were conducted in Se-Al (http://tree.bio.ed.ac.uk/software/seal/), ensuring that reading frame and amino acid alignment within the retained sections were preserved. Finally, the original nucleotide alignment was also passed through the GBlocks program to objectively remove regions of ambiguous alignment (9255 nt). Only one sequence per virus strain was used, with the exception of CxFV, for which strains from Japan, Mexico and Uganda were used due to their wide geographical distribution and different mosquito ‘host’ species. In the case of CFAV, the strain isolated from a natural mosquito population (CFAV Culebra) was used in preference to that isolated from a laboratory cell culture. The rdp, geneconv and Bootscan methods were all employed within the rdp3 package (Martin *et al.*, 2010). Bootscan analyses were conducted with window sizes of 50, 100, 200 and 500 nt.

### Tests for co-divergence of ‘insect-specific’ flaviviruses and vectors

To test the level of congruence between the phylogeny of the ‘insect-specific’ flaviviruses and their mosquito hosts, only well-characterized virus taxa isolated in cell culture and with full ORF sequence available were included. Similarly, only those mosquito ‘hosts’ that have been incriminated more than once for a given flavivirus and/or via both molecular and morphological identification, preferably from individual mosquitoes rather than from pools, were used, as shown in Table 1.

**Table 1.** ‘Insect-specific’ flaviviral strains and mosquito association tested for co-divergence

Virus strain	Vector
NAKV	*Mansonia africana*
CxFV_Tokyo	*Culex pipiens*
CxFV_Japan03	*Culex pipiens*
CxFV_HOU24518	*Culex quinquefasciatus*
CxFV_Iowa07	*Culex pipiens*
CxFV_Mexico07	*Culex quinquefasciatus*
CxFV_Uganda08	*Culex quinquefasciatus*
QBV	*Culex quinquefasciatus*
CFAV_Culebra	*Stegomyia aegypti*
CFAV_Cammisa	*Stegomyia aegypti*
AeFV	*Stegomyia albopicta*
KRV_SR75	*Neomelaniconion mcintoshi*
KRV_SR82	*Neomelaniconion mcintoshi*
DENV1	*Stegomyia aegypti*

**Fig. 1.**
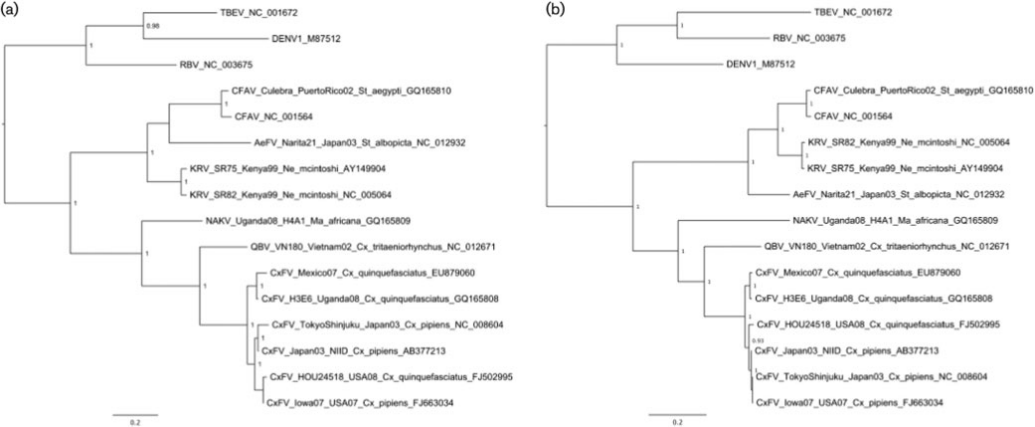
Bayesian phylogenies of (a) the NS5 and (b) the NS3 ‘insect-specific’ focus nucleotide datasets. Only posterior probabilities of ≥0.9 are included. Both trees are midpoint-rooted. Bars, 0.2 substitutions per site.

**Fig. 5.**
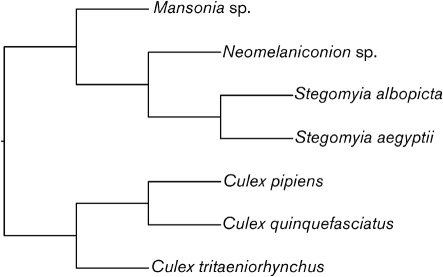
Simple topology used for mosquito species included in co-divergence tests. The two *Culex* species are sister species, which are sometimes regarded as subspecies of *Cx*. *pipiens*. Their sister relationship is not in doubt. *Cx. tritaeniorhynchus* is placed as sister to the *pipiens*+*quinquefasciatus* clade (St John, 2007; Rossi & Harbach, 2008).

To determine the level of congruence between the virus and mosquito phylogenies, reconciliation analyses were conducted in the program TreeMap v2.0 (Charleston & Robertson, 2002). TreeMap maps the nodes of a resolved virus phylogeny onto fixed, associated nodes on the ‘host’ (in this case mosquito) phylogeny and is the most statistically rigorous co-divergence test currently available (Ramsden *et al.*, 2009). For the flaviviruses, the NS5 ‘insect-specific focus’ topology as shown in Fig. 1 (see Phylogenies of the ‘insect-specific’ flaviviruses) was employed. For the mosquitoes, there are currently no published molecular or morphological phylogenies containing all of the associated genera. The few taxon-limited molecular studies that have been conducted do not include *Mansonia* or *Neomelaniconion*. Hence, a topology was constructed based on the phylogenetic studies of Harbach & Kitching (1998), St John (2007), Rossi & Harbach (2008) and Reinert *et al.* (2009), as shown in Fig. 5.

All potentially optimal combinations (POpt) of host switches, duplications, losses and co-divergence events (CEs) were considered as hypothetical scenarios to explain the observed phylogeny of the ‘insect-specific’ flaviviruses given that of their mosquito ‘hosts’. In addition to testing the ‘insect-specific’ viruses (as per Fig. 1) against ‘host’ phylogenies (as per Fig. 5), equivalent tests were also conducted for the *Stegomyia-* and *Culex*-associated ‘insect-specific’ flaviviruses and their ‘hosts’ alone to investigate whether each group exhibited a different pattern of co-divergence. Significance testing was conducted by creating 200 random viral trees (or 100 in the case of the *Stegomyia-* and *Culex*-specific tests) and mapping these onto the fixed host tree. The proportion of these reconciliations with equal or fewer numbers of non-co-divergence events (NCEs) or the same or greater numbers of CEs, compared with the NS5 viral phylogeny, was calculated. Significance was tested using both a minimum CE requirement and a maximum NCE (switches, duplications and losses) requirement. Our null hypothesis was that the level of congruence is no greater than that expected between randomly generated trees at *P*<0.05 for both the number of CEs and NCEs (i.e. both tests are significant at the *P*<0.05 level). This requirement (i.e. both tests be significant) corrects for the more sensitive nature of the NCE test relative to the more widely used CE test.

## Phylogenies of the ‘insect-specific’ flaviviruses

### The NS5 gene region

For the region encoding the NS5 gene of the ‘insect-specific’ sequences, the dataset comprising the longest available multiple sequence alignment contained 16 taxa and was 918 nt in length. The resulting midpoint-rooted Bayesian MCC phylogeny for the NS5 dataset is shown in Fig. 1(a). In all MAP and MCC phylogenies for the various NS5 ‘insect-specific’ datasets, CFAV is related most closely to AeFV, with the pair forming a sister group to KRV, although posterior support values for the position of AeFV were poor compared with those for all other nodes (i.e. <0.9). Overall, the CFAV+AeFV+KRV clade appeared to form a sister group to the CxFV+QBV+NAKV clade.

**Fig. 2.**
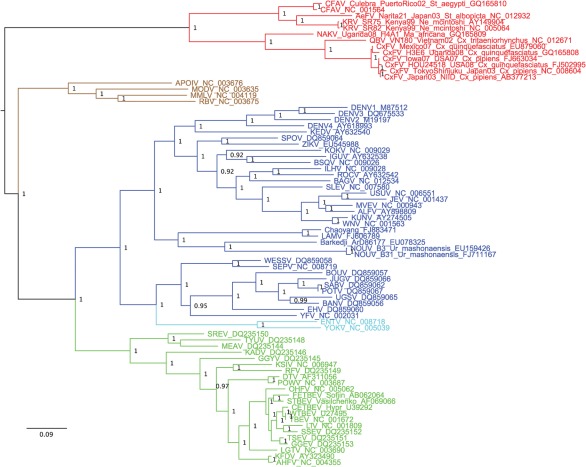
Bayesian phylogeny of the NS5 ‘global genus’ amino acid dataset. Only posterior probabilities of ≥0.9 are included. The tree is midpoint-rooted. Bar, 0.09 substitutions per site. Red, ‘insect-specific’ flaviviruses; brown, NKV flaviviruses; blue, mosquito-borne flaviviruses; light blue, secondary loss flaviviruses; green, tick-borne flaviviruses.

The midpoint-rooted amino acid MCC tree for the ‘global genus’ NS5 dataset is shown in Fig. 2. As with previous studies, the tick- and mosquito-borne flaviviruses appear to form a sister group to the NKV group. As in the ‘insect-specific focus’ NS5 analysis, the insect-specific flaviviruses fall into two clades, with the CFAV+AeFV+KRV clade again forming a sister group to the CxFV+QBV+NAKV clade.

### The NS3 gene region

The MAP and MCC tree topologies were identical for all NS3 ‘insect-specific focus’ datasets (namely full nucleotide, third codon position removed or amino acid alignments). The amino acid MCC tree is shown in Fig. 1(b). Notably, and in contrast to the NS5 gene phylogenies, CFAV and KRV formed a well-supported sister group to AeFV in the NS3 phylogeny. Within the CxFV sequences, there is an indication that sequences from Uganda and Mexico isolated from *Culex quinquefasciatus* may be related more closely to one another than to CxFV sequences isolated in *Cx. pipiens* from Japan and the USA.

For the ‘global genus’ NS3 datasets, the MAP and MCC phylogenies differed from those observed previously in the flaviviruses. The mosquito-borne flaviviruses fell into two clades instead of one in all cases. One clade included YFV (Old World, *Stegomyia*-associated) plus the ‘secondary loss’ Entebbe bat virus and Yokose virus sequences (Kuno *et al.*, 1998). The second clade contained the remaining mosquito-borne flaviviruses, such as DENV (cosmopolitan, *Stegomyia*-associated) and the *Culex*-associated mosquito-borne flaviviruses, forming a sister group to the NKV plus tick-borne flaviviruses (not shown). To investigate this ‘novel’ tree topology further, half of the insect-specific sequences were randomly deleted from the original amino acid NS3 dataset, and analyses were repeated. This restored the original ‘NS3’ topology wherein the NKV and tick-borne viruses formed a sister group to the mosquito-borne clade (similar to the MCC tree for the ORF amino acid dataset; see Fig. 4). This process was repeated 10 times with the same results, suggesting either that (i) alignment error was caused by the introduction of the highly divergent insect-specific sequences or (ii) a segment(s) of the NS3 gene in the YFV group may be related more closely to NS3 gene sequences in the insect-specific flaviviruses.

### The E gene region

**Fig. 3.**
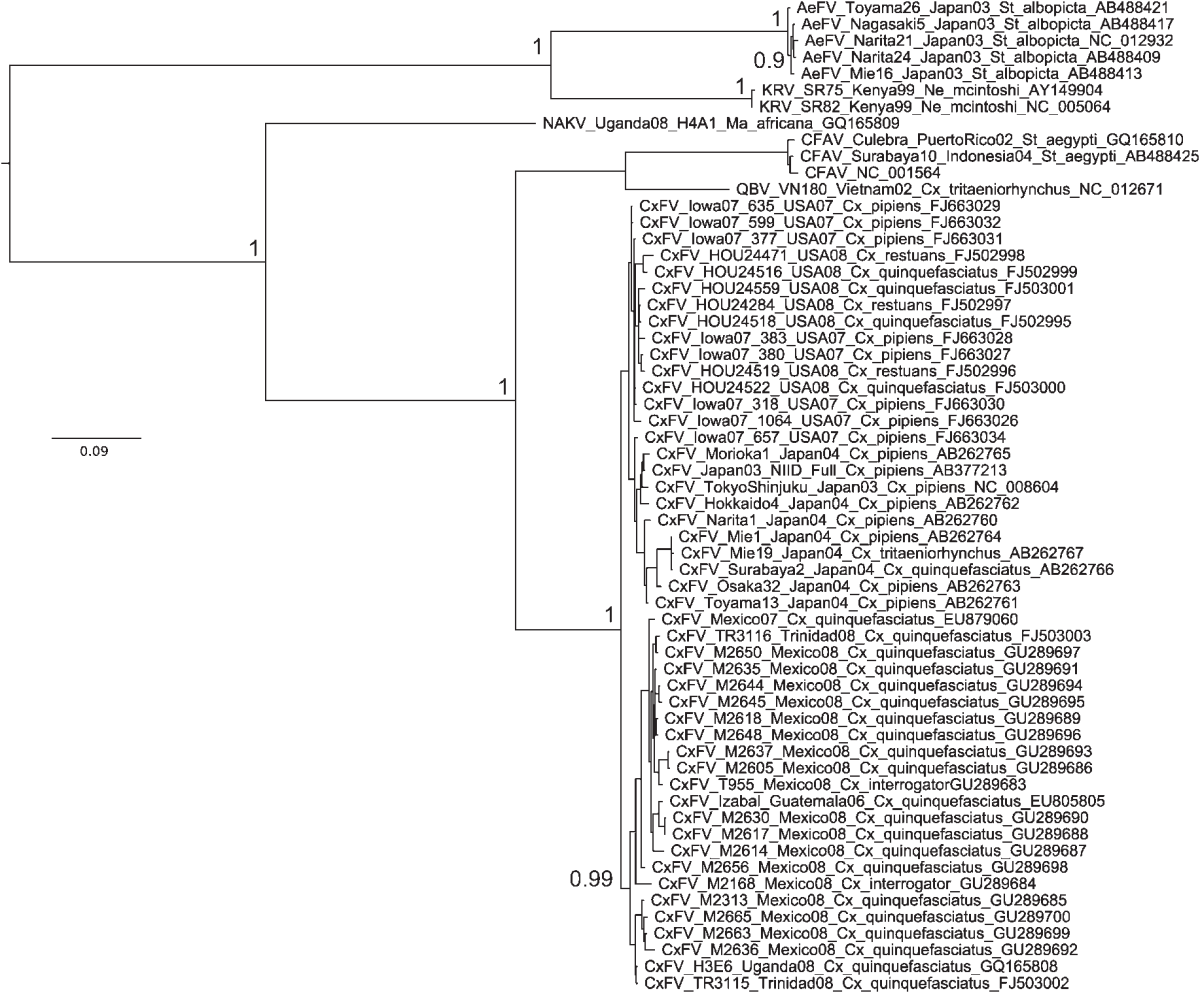
Bayesian phylogeny of the E gene ‘insect-specific’ focus amino acid dataset. Posterior probabilities of ≥0.9 for major nodes only are included for clarity. The tree is midpoint-rooted. Bar, 0.09 substitutions per site.

All E gene insect-specific MAP and MCC tree topologies were identical to the MCC tree for the amino acid dataset (Fig. 3). In all cases, CFAV was related most closely to QBV, forming a sister group to the numerous CxFV sequences and with NAKV basal to all three viruses, whereas KRV and AeFV formed a second, distinct clade of insect-specific flaviviruses. Importantly, the position of CFAV in the E gene phylogenies contrasted strongly with its position in the NS5 and NS3 phylogenies. This is in agreement with previous studies that included a more limited number of ‘insect-specific’ lineages, namely one strain each of CxFV, KRV and CFAV (Hoshino *et al.*, 2007).

Notably, a 14 aa motif present in the E protein of the ‘traditional’ flaviviruses (TABV), thought to be involved in viral envelope fusion with cellular endosomes and entry of viral RNA (de Lamballerie *et al.*, 2002; Roehrig *et al.*, 1989), appeared to be highly conserved in the ‘insect-specific’ flaviviruses, as shown in Table 2. Also, all cysteine residues present in the E gene region of the ‘insect-specific’ sequences analysed were completely conserved.

**Table 2.** Conserved fusion peptide motif in the ‘insect-specific’ flaviviral E gene region shared with the other members of the genus

Flavivirus	Fusion peptide motif
KRV, AeFV	N**RGW**GT**GC**FEW**G**L**G**
NAKV	N**RGW**GT**GC**LEW**G**L**G**
CFAV, CxFV	N**RGW**GT**GC**FKW**G**L**G**
All ‘traditional’ flaviviruses and TABV	D**RG**WXX**GC**XXF**G**K**G**/H

### Polyprotein ORF analyses

**Fig. 4.**
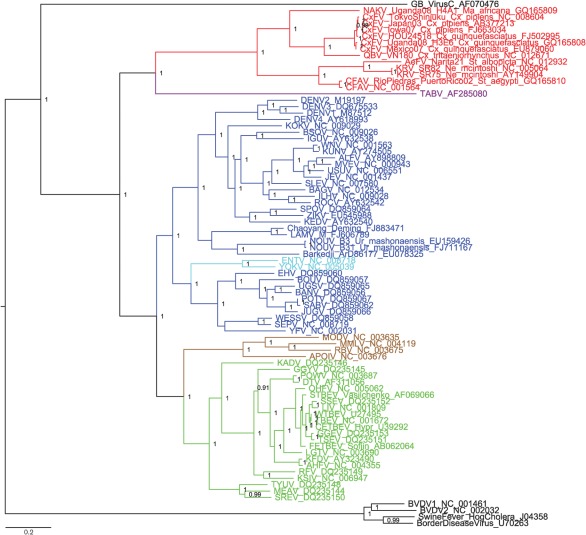
Bayesian phylogeny of the ORF ‘global genus’ amino acid dataset. Only posterior probabilities of ≥0.9 are included. The tree is rooted on GB virus C. Bar, 0.2 substitutions per site. Red, ‘insect-specific’ flaviviruses; brown, NKV flaviviruses; blue, mosquito-borne flaviviruses; light blue, secondary loss flaviviruses; green, tick-borne flaviviruses; purple, TABV.

The MCC tree for the ORF amino acid dataset (including members of the family *Flaviviridae* primarily as outgroup taxa) is shown in Fig. 4. Its general topology was also reflected in the two phylogenies (with and without GBlocks stripping) of the genus *Flavivirus* alone. The ‘traditional’ mosquito-borne flaviviruses (MBFV) formed a sister group to the tick-borne flaviviruses (TBFV) and NKV clades as reported in previous studies (Billoir *et al.*, 2000; Cook & Holmes, 2006). TABV and the insect-specific flaviviruses fall as a sister group to the MBFV+TBFV+NKV group.

### Topology tests

As shown in Supplementary Table S1 (available in JGV Online), results suggest that all NS3 topologies, plus the ORF MCC topology, have significantly lower likelihoods (i.e. fit the data worse) for the NS5 dataset than the best tree inferred by the NS5 dataset. For the NS3 dataset, all the NS5 topologies are worse hypotheses of the evolutionary relationships of the NS3 data than the NS3 topologies, but the ORF topologies are not significantly different.

### Recombination in the ‘insect-specific’ flaviviruses

No recombination was detected in the ORF alignments with either no adjustment (length 10 461 nt) or simple trimming at both ends (9894 nt). In contrast, after removal of regions of ambiguous alignment via GBlocks stripping (9255 nt alignment) or via manual deletion (8835nt alignment), evidence for recombination was detected at positions 238–1948 and 1–1678, respectively (which represented the same region, with differential removal of ambiguous alignment by each deletion method). The GBlocks alignment gave *P*-values <10^−17^ for each of the rdp, geneconv and Bootscan methods, whereas the manual deletion alignment gave *P*-values of <10^−40^ for the same methods. Overall, results suggest that CFAV may contain a fragment of recombinant sequence in the E gene region, with the potential major parent sequence being related to KRV and the potential minor parent being related to QBV. Significantly, these positions represent parts of the region encoding the E gene and include the fusion peptide motif listed in Table 2.

### Tests for co-divergence of ‘insect-specific’ flaviviruses and vectors

We found no significant support for co-divergence at the level of *P*<0.05 for either co-divergence test (requiring that tests using both the number of CEs and NCEs be significant) given the sampled viruses, for either all of the ‘insect-specific’ flaviviruses analysed together or the *Stegomyia-* and *Culex-*isolated ‘insect-specific’ flaviviruses analysed alone (Table 3).

**Table 3.** ‘Insect-specific’ flavivirus–host phylogenetic reconciliation analysis The number of POpt solutions and number of events (CEs and NCEs) for POpt solutions are shown. *P*-values represent the number of randomizations out of 200 that fit within the constraints of the CEs and NCEs respectively (or out of 100 randomizations for the *Stegomyia*- or *Culex*-only tests). Significance was tested using both a minimum CEs requirement and a maximum NCEs requirement (i.e. significance was assigned only if both the min. CE and max. NCE tests had *P*-values <0.05).

Topology analysed/solution	Min. CEs	Significance (*P*) of CEs	Max. NCEs	Significance (*P*) of NCEs
**All insect-specific**				
S1	10	<0.905	16	<0.02
S2	10	<0.905	17	<0.02
S3	10	<0.905	19	<0.06
***Culex* only**				
S1	4	<0.93	9	<0.44
S2	4	<0.93	10	<0.44
***Stegomyia* only**				
S1	6	<0.39	4	<0.01

## Discussion

We present the most comprehensive phylogenetic study of the tentative ‘insect-specific’ members of the genus *Flavivirus* to date. In the majority of the phylogenies generated, the ‘insect-specific’ flaviviruses fell into two clades, with the *Stegomyia-*associated CFAV+AeFV+KRV forming a sister group to the *Culex-*associated CxFV+QBV, plus NAKV from a *Mansonia* species of mosquito. However, in phylogenies generated from the E gene region, CFAV was related most closely to QBV, with evidence for recombination in both the laboratory and natural isolates of CFAV. Results from Shimodaira–Hasegawa (SH) tests of the NS3 and the NS5 gene regions versus ORF topologies for the entire genus suggest that, in common with previous studies and in contrast to NS5, the NS3 dataset and topologies are not significantly different from those inferred by the ORF dataset (Billoir *et al.*, 2000; Cook & Holmes, 2006).

In all of the inferred phylogenies for single gene region and ORF datasets for the ‘insect-specific’ group, there is a strongly supported split between *Culex*- and *Stegomyia*-associated sequences. However, we found no statistical support for host–virus co-divergence in the ‘insect-specific’ flaviviruses based on the data analysed here. These findings are in agreement with the hypothesis that ‘insect-specific’ flaviviruses may have undergone multiple introductions with frequent host-switching and/or potential occasional recombination events, at least in the case of CFAV. This is further supported by the fact that, based on our current knowledge of flaviviral and mosquito evolution, the divergence of some of the relevant mosquito species, such as *St. albopicta* and *St. aegypti* [approx. 34–42 million years ago (mya)], is significantly more ancient than the most recent common ancestor of CFAV and KRV (probably around 3500 ya, and certainly less than 350 000 ya; Crochu *et al.*, 2004). The poor support for the position of AeFV in many of the phylogenies may reflect the fact that this virus (or group of related *Stegomyia*-associated viruses) is more diverse or widespread in nature than other ‘insect-specific’ flaviviruses, and sampling is not taxonomically or geographically sufficient to provide good resolution. CxFV sequences from *Cx. quinquefasciatus* from Uganda and Mexico appear to be related more closely to one another than to CxFV sequences from *Cx. pipiens* from Japan and the USA. Kim *et al.* (2009) also found that North American strains of CxFV were related more closely to Asian strains than to Central American and Caribbean strains. In terms of ‘host’ biology, *Cx. quinquefasciatus*, *Cx. pipiens* and *Cx. tritaeniorhynchus* are all probably African in origin and were spread to Asia via human activity. *Cx. quinquefasciatus* and *Cx. pipiens* are cosmopolitan in distribution and exist as sister species that often hybridize in non-indigenous regions. CxFV appears to have been introduced multiple times to the New World in association with *Culex* species. NAKV was isolated from a *Mansonia* mosquito in Uganda. The morphology of *Mansonia* mosquitoes suggests that they may be related more closely to the Aedini than to *Culex* species. *Neomelaniconion mcintoshi*, the species from which KRV was isolated, is an aedine mosquito related more closely to *Stegomyia* than to *Culex* mosquitoes (Harbach & Kitching, 1998; Reinert *et al.*, 2009).

In the wider ‘global genus’ analyses, which included the divergent TABV and members of the family *Flaviviridae* in some datasets, the ‘traditional’ MBFV in general form a sister group to the TBFV and NKV clades as in previous studies (Billoir *et al.*, 2000; Cook *et al.*, 2006). TABV plus the ‘insect-specific’ flaviviruses then fall as a sister group to the MBFV+TBFV+NKV group. Previous phylogenetic analyses based on the NS3 or NS5 gene regions only or the entire polyprotein sequence suggested that the TABV lineage, which appears to replicate in mammalian cells only, is basal to the genus *Flavivirus* (Hoshino *et al.*, 2007, 2009; Lobo *et al.*, 2009). Kuno *et al.* (1998) and Gaunt *et al.* (2001) suggested that the NKV group evolved from the distantly related flaviviruses at the root of the NS5 tree and subsequently the tick-borne and then the mosquito-borne groups evolved from the NKV group, thus postulating that flaviviral association with ticks was more primitive than the association with mosquitoes and that vector-borne transmission was an acquired trait. Our results do not support a basal position of the NKV flaviviruses within the genus *Flavivirus* (Kuno & Chang, 2005), although, significantly, in Fig. 4 we have made the assumption of rooting on the highly divergent GB virus C. de Lamballerie *et al.* (2002) found minimal differences in likelihoods between trees in which (i) TABV was represented as the sister taxon to a group containing the taxonomically recognized viruses in the genus *Flavivirus* plus the ‘insect-specific’ flaviviruses, (ii) TABV was related more closely to the recognized viruses in the genus *Flavivirus* than to the ‘insect-specific’ flaviviruses or (iii) TABV formed a distinct clade falling together with the ‘insect-specific’ flaviviruses. At that time, the ‘insect-specific’ flaviviruses were represented only by CFAV. The phylogenetic analyses conducted here seem to support option (iii), in which the ‘insect-specific’ flaviviruses plus TABV are related more closely to one another than to the MBF+TBF+NKV group, but this requires further study, ideally using additional taxa and methods for dealing with highly divergent sequences.

In a number of the NS5 ‘sliding window’ phylogenies (not shown here due to short alignment length), some sequences containing stop codons, presumably non-functional, fell with apparently functional sequences. These sequences were detected via RT-PCR in pools of mosquitoes, and virus isolation attempts were unsuccessful (Calzolari *et al.*, 2010). As noted by the authors at the time, this may reflect the fact that either (i) all sequences from that source actually comprise virus integrations into the mosquito genome rather than active virus infection, or (ii) virus was actually present, but was rendered non-viable during the repeated freeze–thawing process. If, indeed, viable, replicative viruses harbouring sequences containing stop codons were present (or are discovered in future studies), we hypothesize that the function of the ‘insect-specific’ NS5 flaviviral protein may be maintained via ribosomal frameshifting, as already documented for the NS2A/NS2B of the ‘insect-specific’ flaviviruses (Firth *et al.*, 2010). Visual inspection of the NS5 ‘non-functional’ sequences in question indeed shows that a frameshift of +1 would restore function in the majority of cases. Interestingly, manual inspection also provides preliminary evidence of overlapping coding sequence (CDS) in CxFV, where a frameshift towards the 3′ terminus of the NS5 gene produces a viable 70 aa sequence. This is comparable in size to the 52-codon *foo* (flavivirus overlapping ORF) in the NS1–NS2A of JEV (Firth & Atkins, 2009; Firth *et al.*, 2010) and the 300-codon overlapping CDS in the ‘insect-specific’ flaviviruses in the NS2A/NS2B, termed *fifo* (Firth *et al.*, 2010). An additional, perhaps more likely, possibility is that these defective viruses may be rescued by complementation, in which defective genomes are rescued through the parasitism of functional proteins from wild-type viruses, as documented in DENV-1 (Aaskov *et al.*, 2006).

We provide preliminary evidence for a recombinant history for CFAV, although this clearly needs to be verified as this process was only apparent in some datasets. Interestingly, the relevant partial sequence identified as a potential recombinant region appears to include the fusion peptide protein. Phylogenetic analysis of partial genome sequences has suggested homologous recombination between closely related strains of DENV (Tolou *et al.*, 2001; Twiddy & Holmes, 2003; Uzcategui *et al.*, 2001) and JEV and St Louis encephalitis virus (Gould *et al.*, 2004); recombination between flavivirus genomes has also been documented in the laboratory under experimental conditions, albeit producing viruses with an impaired-growth phenotype (Taucher *et al.*, 2010). Recent studies using DENV have suggested that C6/36 cells may exhibit a dysfunctional RNA interference response to infection (Brackney *et al.*, 2010; Scott *et al.*, 2010). We may hypothesize that C6/36 *St. albopicta* cells may also exhibit a potentially different and/or dysfunctional response to infection with ‘insect-specific’ flaviviruses, and a relatively inefficient immune response in wild *St. albopicta* mosquitoes could have provided the circumstances for co-infection with more than one virus and recombination in CFAV.

We recognize that all hypotheses are highly speculative because the ‘insect-specific’ flaviviruses are likely to be significantly undersampled at present. Further, and crucially, precise data are required to test our conclusions. First, data regarding the host species for the ‘insect-specific’ flaviviruses are limited because, for many of the publicly available ‘insect-specific’ flavivirus sequences, molecular identification of the mosquito species and/or de-pooling to test individual specimens was not conducted. This is despite the fact that many *Stegomyia* and *Culex* mosquitoes may exist as sibling (cryptic) species complexes and/or can only be identified reliably via dissection of the male genitalia. Also, few molecular markers have been documented that provide appropriate resolution across the tribes Aedini and Culicini, which, respectively, include *Stegomyia* and *Culex* mosquitoes. Second, our review of all ‘insect-specific’ studies conducted to date shows that the accuracy and availability of flavivirus data are limited due to (i) frequent lack of isolation in cell culture and/or sufficient tests to distinguish integrations of flavivirus-like sequence in mosquito genomes from viable viruses, and (ii) differing taxonomic coverage across species and/or gene regions. For example, flaviviral sequences have been detected in pools of mosquitoes or phlebotomine sandflies, but isolation in cell culture has not been possible due to the use of guanidium thiocyanate for nucleic acid extraction (Aranda *et al.*, 2009; Moureau *et al.*, 2010) or repeated freeze–thaw cycles (Calzolari *et al.*, 2010). Some tentative ‘insect-specific’ flavivirus sequences obtained via RT-PCR only have not been submitted to public databases due to insufficient sequence length. In many instances, it is not proven whether the sequences are of flavivirus origin or result from integrations. For example, some sections of apparently ‘insect-specific’ flaviviral sequence may have been amplified from cultures in which carry-over of mosquito DNA integrations from original pool inoculum and/or from the C6/36 cell cultures themselves cannot be discounted. In addition, and potentially confounding results further, our previous experiments have shown that DNA sequences of ‘insect-specific’ flaviviruses can be detected during early infection (Cook *et al.*, 2006). In numerous cases, (i) electron microscopy of virion particles, (ii) full flaviviral genome sequencing and/or genome walking to determine whether flanking regions are of insect or viral origin or (iii) the use of DNase and/or a reverse-transcription negative control has not been conducted thoroughly to determine the absolute presence of a virus (Morales-Betoulle *et al.*, 2008; Pabbaraju *et al.*, 2009; Roiz *et al.*, 2009; Sánchez-Seco *et al.*, 2010; Farfan-Ale *et al.*, 2009, 2010; Bolling *et al.*, 2011).

The following characteristics are determined by the ICTV as bringing viruses together in the genus *Flavivirus*: virion properties, nucleic acid and protein characteristics, genome organization, replication strategy and antigenic properties. Importantly, biological properties are also taken into consideration, including host range, transmission mode and vector relationships, geographical distribution and association with disease. With this in mind, the significant similarity of ‘insect-specific’ flaviviruses to one another with regard to genome size and organization, and sequence identity in certain structural and functional components of the viral proteins, appears to support the designation of these viruses as a particular group within the flaviviruses, but the requisite data for many potential members have not been provided. To date, for those novel strains that have been characterized sufficiently, >84 % pairwise sequence identity has been used as a criterion for species delineation (Kuno *et al.*, 1998). Taken together, to construct an accurate foundation for the study of novel flaviviruses, it is advisable (i) to identify mosquitoes via integrated morphological and molecular means, (ii) to homogenize mosquitoes individually before pooling and retain a proportion of homogenate at −80 °C without repeat freeze–thaw cycles and (iii) to conduct screening via both RT-PCR and cell-culture methods. Molecular biology screening should incorporate DNase- and reverse transcriptase-free control tests for integrations plus full sequencing of the virus via genome walking to check for the presence of insect sequence at either the 5′ or 3′ end of potential integrations. In addition, many novel strains are likely to be discovered in natural tick populations in future. It is becoming increasingly necessary to clarify a basis for the taxonomy of the ‘insect-specific’ flaviviruses. In particular, at present there exists potential for inaccuracy and/or confusion caused by the naming of ‘insect-specific’ viruses according to mosquito host, and a lack of formal criteria to define species versus strains within the group.

Overall, we have shown that the insect-specific and ‘traditional’ flaviviruses appear to form sister groups, which are both highly divergent from these other members of the family *Flaviviridae*. As a consequence, the screening of field samples from a wide range of mammals using pan-flaviviral real-time RT-PCR may also reveal further viruses and provide essential information regarding deep-level evolution in the family.
